# Effect of varying quantities of lean beef as part of a Mediterranean-style dietary pattern on lipids and lipoproteins: a randomized crossover controlled feeding trial

**DOI:** 10.1093/ajcn/nqaa375

**Published:** 2021-04-07

**Authors:** Jennifer A Fleming, Penny M Kris-Etherton, Kristina S Petersen, David J Baer

**Affiliations:** Department of Nutritional Sciences, The Pennsylvania State University, University Park, PA, USA; Department of Nutritional Sciences, The Pennsylvania State University, University Park, PA, USA; Department of Nutritional Sciences, The Pennsylvania State University, University Park, PA, USA; Department of Nutritional Sciences, Texas Tech University, Lubbock, TX, USA; USDA/ARS/BHNRC Food Components and Health Laboratory, Beltsville, MD, USA

**Keywords:** Mediterranean diet, lean beef, lipids, lipoproteins, cardiovascular disease

## Abstract

**Background:**

It remains unclear whether red meat consumption is causatively associated with cardiovascular disease (CVD) risk, and few randomized controlled studies have examined the effect of incorporating lean beef into a healthy dietary pattern.

**Objectives:**

To evaluate the effects of a Mediterranean (MED) diet (carbohydrate 42%, protein 17%, fat 41%, SFAs 8%, MUFAs 26%, PUFAs 8%) with 14 (MED0.5; 0.5 oz), 71 (MED2.5; 2.5 oz), and 156 (MED5.5; 5.5 oz) g/d/2000 kcal lean beef compared with an average American diet (AAD; carbohydrate 52%, protein 15%, fat 33%, SFAs 12%, MUFAs 13%, PUFAs 8%) on lipid and lipoprotein concentrations, particle number, and size.

**Methods:**

This was a multicenter, 4-period controlled feeding, randomized crossover study. Fifty-nine generally healthy males and females (BMI 20–38 kg/m^2^; age 30–65 y) consumed each diet for 4 wk with a ≥1-wk washout between the diets. Fasting blood samples were collected at baseline and at the end of each 4-wk period. Lipid subfractions were measured by NMR.

**Results:**

Compared with the AAD, all 3 MED diets decreased LDL cholesterol (MED0.5: −10.3 mg/dL; 95% CI: −5.4, −15.7 mg/dL; MED2.5: −9.1 mg/dL; 95% CI: −3.9, −14.3 mg/dL; MED5.5: −6.9 mg/dL; 95% CI: −1.7, −12.1 mg/dL; *P* < 0.0001). All MED diets elicited similar reductions in total LDL particle number compared with baseline (*P* < 0.005); however, significant decreases only occurred with MED0.5 (−91.2 nmol/L; 95% CI: −31.4, −151.0 nmol/L) and MED2.5 (−85.3 nmol/L; 95% CI: −25.4, −145.2 nmol/L) compared with AAD (*P* < 0.003). Compared with the AAD, non-HDL cholesterol (*P* < 0.01) and apoB (*P* < 0.01) were lower following the 3 MED diets; there were no differences between the MED diets. All diets reduced HDL-cholesterol and HDL particle number from baseline (*P* < 0.01).

**Conclusions:**

Lipid and lipoprotein lowering was not attenuated with the inclusion of lean beef in amounts ≤71 g (2.5 oz)/d as part of a healthy low-saturated-fat Mediterranean-style diet.

This study is registered at clinicaltrials.gov as NCT02723617.

## Introduction

Epidemiological studies suggest that higher red meat consumption is associated with increased risk of cardiovascular disease (CVD) (1, 2). However, causation cannot be inferred from these analyses and it remains unclear whether intake of red meat per se increases the risk of CVD or if these associations are because of other dietary and lifestyle behaviors that co-occur with red meat consumption. Importantly, in these epidemiological studies the isolated effect of red meat is difficult to disentangle from the rest of the diet. Furthermore, the definition of red meat is heterogeneous, and often unprocessed and processed red meat are examined as a single red meat exposure. A recent meta-analysis of randomized controlled trials reported that the effect of red meat on CVD risk factors is dependent on the comparison diet and the dietary substitutions made to incorporate red meat into the diet (3). Current recommendations are focused on dietary patterns because nutrients, foods, and food components are not consumed in isolation, and the totality of the diet has a greater effect on health than the individual components, hence investigation of red meat consumption as part of well-defined dietary patterns is needed.

A growing clinical evidence base suggests that lean, unprocessed red meat can be included as part of a heart-healthy eating pattern without adversely affecting CVD risk factors (4–8). In a randomized crossover study, consumption of a low-saturated-fat (7%) Mediterranean diet with 500 g/wk (2.5 oz/d) lean unprocessed red meat reduced total cholesterol and LDL cholesterol after 5 wk of controlled feeding (8). This finding provides some evidence that including greater amounts of lean unprocessed red meat than recommended by the Mediterranean Diet Pyramid (<120 g/w) (9) does not attenuate lipid and lipoprotein lowering as part of a cholesterol-lowering diet. However, it is not clear whether inclusion of lean unprocessed red meat in quantities greater than recommended by the Mediterranean Diet Pyramid (while meeting Mediterranean diet food-based recommendations) dose-dependently affects lipids and lipoproteins.

The primary aim of this controlled feeding trial was to examine the dose–response effect of including lean beef [14, 71, 156 g/d/2000 kcal (0.5, 2.5, 5.5 oz/d/2000 kcal)] as part of a healthy Mediterranean-style (MED) diet on blood lipids, lipoproteins, and apolipoproteins compared with an average American diet (AAD) containing ∼71 g (2.5 oz) beef/d/2000 kcal. In addition, we examined the role of plasma proprotein convertase subtilisin/kexin type 9 (PCSK9) as a potential mechanism by which a MED diet with different quantities of lean beef lowers LDL cholesterol. We hypothesized that all 3 MED diets would elicit greater improvements in lipids and lipoproteins compared with the AAD in generally healthy adults, and that these benefits would be similar across all diets based on their shared macronutrient profile.

## Methods

### Experimental design

**FIGURE 1:**
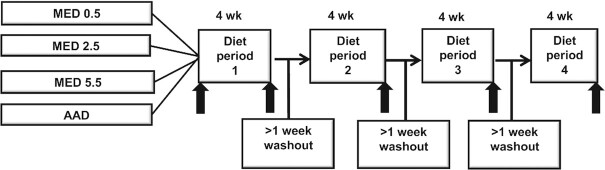
Study design. Clinical assessments were conducted across 2 consecutive days. AAD, average American diet; MED, Mediterranean-style eating pattern used in the study; MED0.5, MED diet with 14 g (0.5 oz) per day of lean beef; MED2.5, MED diet with 71 g (2.5 oz) per day of lean beef; MED5.5: MED diet with 156 g (5.5 oz) per day of lean beef based on a 2000-kcal diet.

A 4-period, randomized, crossover, controlled-feeding study was conducted at 2 centers: Penn State University and USDA-Beltsville Human Nutrition Research Center. The 3 MED test diets included the following: *1*) 14 g (0.5 oz) beef/d/2000 kcal (MED0.5), which represents the amount recommended in the Mediterranean Diet Pyramid (9); *2*) 71 g (2.5 oz) beef/d/2000 kcal (MED2.5), which represents current consumption patterns in the United States (10); and *3*) 156 g (5.5 oz) beef/d/2000 kcal, which represents an amount previously shown to elicit heart health benefits when consumed as part of a Dietary Approaches to Stop Hypertension (DASH)-style diet (4). Participants were randomly allocated to 1 of 12 diet sequences to ensure that diets were assigned in a balanced order. The block randomization code was generated by an independent USDA staff member using an orthogonal Latin-square design with 5 blocks (5 replicates) and 12 sequences per block. Participants received each diet for 4 wk with a washout period of ≥1 wk between diet periods in which they resumed their self-selected diet (**Figure 1**). The participants were not blinded; however, the study coordinator, investigators, analysts, and statisticians were blinded for purposes of outcome assessment and statistical analysis. The Institutional Review Board at the Pennsylvania State University and MedStar Health Research Institute (for Beltsville Human Nutrition Research Center) approved the study protocol before the initiation of the study and all participants provided written informed consent. The trial is registered at clinicaltrials.gov (identifier: NCT02723617).

### Study population

Nonsmoking individuals with a BMI >20 and <40 kg/m^2^, aged 30–70 y, were recruited between October 2016 and November 2017 from the State College (PA) and Beltsville (MD) areas. Individuals were required to make daily food pick-ups therefore recruitment was limited to the communities surrounding these areas. Exclusion criteria included: triglycerides >350 mg/dL; HDL cholesterol <15th percentile of US population (males <37 mg/dL, females <44 mg/dL) (11); fasting glucose >126 mg/dL; blood pressure >160/100 mmHg. Individuals prescribed blood pressure–lowering medications were eligible if they met the specified blood pressure range of <160/100 mmHg and had been on a stable medication dose for ≥6 mo. Individuals with a history of kidney disease, liver disease, gout, untreated or unstable hyper- or hypothyroidism, cancer, gastrointestinal disease, pancreatic disease, other metabolic diseases or malabsorption syndromes, or CVD were not eligible. Use of cholesterol-lowering medication or refusal to discontinue intake of putative cholesterol-lowering supplements (psyllium, fish oil capsules, soy lecithin, niacin, fiber, flax, and phytoestrogens) were also exclusion criteria. Additional exclusion criteria included vegetarianism or other dietary practices that were inconsistent with the test diets; weight change of ≥10% of body weight within 6 mo prior to enrolling in the study; and females who were pregnant, lactating, planning to become pregnant, or who had given birth in the past year. Participants were required to maintain weight and physical activity levels.

### Dietary interventions

Participants consumed a controlled weight maintenance, full-feeding diet with a fixed macronutrient composition that varied only between the MED diets (41% fat, 42% carbohydrate, 17% protein) and the AAD (33% fat, 52% carbohydrate, and 15% protein). Study diets were prepared in the metabolic kitchen facility located at each site and included provision of 3 meals and 2 snacks daily using a 7-d rotating menu for the complete duration of each 4-wk intervention period. Energy requirement was calculated using the Harris–Benedict equation and adjusted for self-reported exercise. Body weight was measured Monday through Friday before breakfast (Beltsville) or at food pick-up appointments (Penn State). The 4 intervention diets included: *1*) a MED diet (MED0.5) including 14 g (0.5 oz)/d lean beef; *2*) a fatty acid–matched MED diet (MED2.5) containing 71 g (2.5 oz)/d lean beef; *3*) a fatty acid–matched MED diet (MED5.5), with 156 g (5.5 oz)/d lean beef; and *4*) an AAD. The amount of lean beef consumed was based on the calculated energy requirements of the participants, with a 2000-kcal diet providing 14, 71, and 156 g (0.5, 2.5, and 5.5 oz)/d for the MED0.5, MED2.5, and MED5.5, respectively.

Compliance during the trial was monitored based on daily and weekly questionnaires asking about the consumption of study and nonstudy foods and beverages and daily weigh-ins. Participants were instructed to consume only the foods provided and to limit their consumption of alcohol (≤2 drinks/wk) and caffeinated beverages (<1180 mL/d, or <40 oz). At the Penn State site, participants could consume their meals on-site (Monday-Friday) or have their meals prepared and packed for off-site consumption. At the Beltsville site, participants consumed their breakfast and dinner on-site (Monday-Friday), and consumed their lunch off-site. At both sites, weekend meals and snacks were packaged for off-site consumption.

Menus were developed using FOOD PROCESSOR (ESHA Research) and the nutrient content of the diet was analyzed to verify macronutrient composition and assure protocol accuracy. In brief, homogenized samples of each menu across 2 calorie levels were analyzed by Covance Laboratories, Inc. A chemical analysis of the nutrient composition of the test diets is presented in **Table 1**.

**TABLE 1:** Nutrient targets and chemical analysis of test diets (based on 2000 kcal/d) prepared at the Penn State University (PSU) and USDA research facilities^1^

	Nutrient targets	MED0.5		MED2.5		MED5.5		Nutrient targets	AAD	
	Med diets	PSU	USDA	PSU	USDA	PSU	USDA	AAD	PSU	USDA
Protein, % E	17	19.7	17.7	19.6	18.2	18.8	19.6	15	17.4	16.5
Carbohydrate, % E	42	46.7	40.7	44.6	40.4	42.2	38.4	52	56.2	50.0
Fat, % E	41	40.8	41.6	44.7	41.3	43.1	42.0	33	34.0	33.5
SFA, % E	8	6.5	7.4	7.1	7.5	7.8	8.8	12	10.0	10.8
MUFA, % E	26	24.0	23.0	25.0	23.1	22.7	21.8	13	11.9	13.3
PUFA, % E	8	7.4	7.8	7.2	7.1	6.8	6.6	8	6.0	4.4
ALA, g	1.5	1.69	1.48	1.58	1.31	1.54	1.17	1.5	1.28	1.03
Marine n–3, g	0.5	0.32	0.21	0.28	0.09	0.27	0.08	<0.1	0.25	0.10
Cholesterol,^2^ mg	<300	—	—	—	—	—	—	<300	—	—
Sodium,^2^ mg	<2300	—	—	—	—	—	—	∼3500	—	—
Beef		14 g (0.5 oz)		71 g (2.5 oz)		156 g (5.5 oz)			∼2.5 oz	

1 On the basis of 2000 kcal/d. Average across a 7-d menu cycle. Values were determined by chemical analysis (Covance Laboratories, Inc.). AAD, average American diet; ALA, α-linolenic acid; MED, Mediterranean-style eating pattern used in the study; MED0.5, MED diet with 14 g (0.5 oz) per day of lean beef; MED2.5, MED diet with 71 g (2.5 oz) per day of lean beef; MED5.5, MED diet with 156 g (5.5 oz) per day of lean beef based on a 2000-kcal diet. % E, percentage of total energy.

^2^Values were determined using FOOD PROCESSOR (ESHA Research).

The Mediterranean-style diet used in this study was representative of the Mediterranean diet described by Fundación Dieta Mediterránea (https://dietamediterranea.com/en/nutrition/) and consistent with US Dietary Guidelines for dietary SFAs and sodium (12). The 3 MED diets were macronutrient matched (∼17% protein, 42% carbohydrate, 41% fat) and contained similar foods with the exception of the amount of beef included and other protein equivalents. Each of the MED diets included 196-g (7-oz) equivalents of protein, of which 14, 71, or 156 g came from beef and the remainder from fish, poultry, pork, nuts, eggs, and legumes. All MED diets provided 250 mg/d EPA and DHA by varying the type of fish provided on each test diet. In addition, all MED diets contained <300 mg/d cholesterol, and <2300 mg/d sodium.

All of the MED diets included olive oil (26–32 g, or ∼2 tbsp) as the predominant fat and provided 3–6 servings of fruit daily and ≥6 servings of vegetables daily (on a 2000-kcal diet). The MED0.5 and 2.5 provided similar amounts of plant-based proteins (i.e., legumes and nuts) whereas lean beef replaced these items in the MED5.5. Total number of servings varied slightly to maintain a consistent protein level (17% of total kilocalories) across the experimental diets. The food-based dietary pattern comparison and one day sample menu appear in Supplementary Tables 1 and 2.

The 14-point Mediterranean Diet Assessment Scale constructed by Martinez-Gonzalez et al. (13) was used to assess the level of adherence to a traditional Mediterranean diet of each of the 4 test diets. A value of 0 or 1 was assigned to each of 14 dietary components. For a beneficial component (olive oil, vegetables, legumes, fruits and nuts, and fish), if the recommended consumption value for the test diet was below the reference criterion it was assigned a value of 0, and if it was at or above the criterion it was assigned a value of 1. For a component presumed to be detrimental (meat, soda, baked goods, and high-fat dairy products), the test diet with a recommended consumption value below the reference criterion was assigned a value of 1, and one above the median was assigned a value of 0. The total Mediterranean diet score ranged from 0 (minimal adherence to the traditional Mediterranean diet) to 14 (maximal adherence).

The lean and extra-lean beef cuts used were purchased from local grocery stores. The USDA defines “extra-lean” beef as containing <5 g/100 g total fat, <2 g/100 g SFA, and <95 mg/100 g cholesterol, and “lean” beef is defined as <10 g/100 g total fat, 4.5 g/100 g SFA, and 95 mg/100 g cholesterol (14). On a selected basis, some higher-fat cuts of beef were used; however, this was done such that total beef consumption on average, met the lean beef definition (because some extra-lean cuts were used). Beef was incorporated into meals in a manner that reflected typical consumption patterns of consumers. For example, 28 g of lean beef in a chili dish, 56 g in a lean beef sandwich or salad, 84 g in a fajita dish, and 112 g in a steak meal.

### Clinical visits and blood sample collection

Blood samples were collected on 2 consecutive days at baseline (start of study) and at the end of each diet period. For the 48 h prior to each collection, participants were told to refrain from alcohol consumption and the use of anti-inflammatory medications. For the 24 h prior, they were asked not to engage in vigorous exercise, and not to consume any food or drink (except water) for the 12 h before their visit. Serum and plasma aliquots were collected and stored at −80°C until time of analysis. This article will present the results of our primary outcomes, lipids and lipoproteins; secondary outcomes will be published in a future article.

### Lipids and lipoproteins

Serum total, HDL, and LDL cholesterol, and triglyceride concentrations were determined by enzymatic procedures using a Vitros Clinical Chemistry Analyzer (VITROS 5,1; Ortho-Clinical Diagnostics, Inc.). Serum apoAI and apoB were measured by immunoturbidimetric assay (VITROS 5,1 to Ortho-Clinical Diagnostics, Inc.). PCSK9 concentrations were measured using a microfluidic platform (Ella; ProteinSimple). Lipid and lipoprotein concentrations and PCSK9 were measured at the USDA site.

Lipoprotein particle number and size were measured by a proton nuclear magnetic resonance spectroscopy assay (NMR; LabCorp). The NMR analysis was conducted according to the method described by Jeyarajah et al. (15).

### Statistical analysis

A sample size of 60 participants (*n* = 30 per site) was determined based on LDL cholesterol. The sample size was estimated to detect a 5% change in LDL cholesterol (assuming a mean LDL cholesterol of 120 mg/dL in the recruited cohort) with the following assumptions: power of 0.9, α of 0.05, expected between-diet SD of 13 mg/dL, and a 2-tailed test. Based on these assumptions, a sample size of 52 was considered sufficient to test the primary LDL cholesterol hypothesis, the change in LDL cholesterol on the experimental diets compared with the AAD. However, this sample size was increased to account for an expected dropout rate of ∼15%.

All statistical analyses were performed using SAS 9.4 (SAS Institute, Inc.). Using PROC UNIVARIATE the residuals for each variable were analyzed to assess normality as well as visual inspection of distributions (histograms and stem and leaf plots), skewness value, and Shapiro–Wilk *P* value. Logarithmic transformations were used for nonnormally distributed variables. The analytic plan was designed a priori and described a mixed-effects model for analysis of the data for repeated measurements (PROC MIXED). All data were analyzed in a manner consistent with an intention-to-treat approach. Available data from every randomly assigned participant were included in the analyses. Data from participants who withdrew from the study were included when endpoint measures were obtained. The mixed-models procedure does not perform listwise deletion and preserves the df, thus it allows for inclusion of participants with ≥1 missing data point in the analyses. For each variable, the mean of 2 sample measurements taken at the end of each feeding period was analyzed. For all models, baseline value, sex, treatment order, and site were included as covariates, subject was included as a random effect, and diet was a fixed effect. Model covariance structures were based on optimizing fit statistics (evaluated as lowest Bayesian Information Criterion). The mixed models procedure (PROC MIXED) was used to test the main effects of diet, period, and their interaction on outcome measures. For each variable, the following analyses were performed: a comparison of means following each diet, the change from baseline following each diet (calculated by subtracting baseline values from endpoint values) as well as a comparison of the change from baseline between the test diets. Values are reported as means ± SEM. To account for the number of primary outcomes, statistical significance for the main effect of diet was set at *P* < 0.003 (Bonferroni adjusted) to reduce the risk of type 1 statistical errors. Where a significant main effect of diet was detected, Tukey–Kramer adjusted *P* values were used for all post hoc pairwise comparisons (*P* < 0.05).

## Results

**FIGURE 2:**
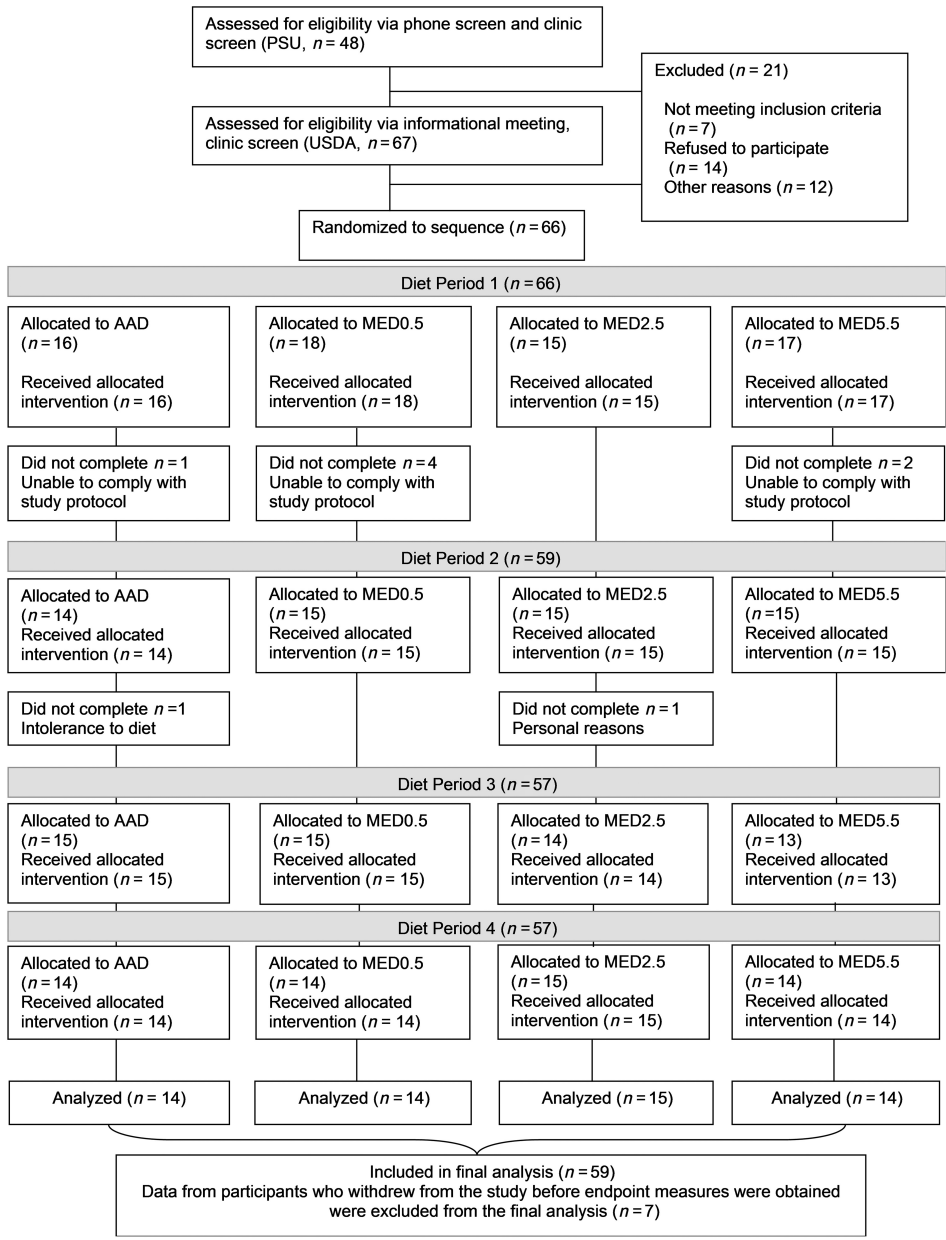
CONSORT diagram showing the flow of participants through each stage of the randomized trial. AAD, average American diet; MED, Mediterranean-style eating pattern used in the study; MED0.5, MED diet with 14 g (0.5 oz) per day of lean beef; MED2.5, MED diet with 71 g (2.5 oz) per day of lean beef; MED5.5: MED diet with 156 g (5.5 oz) per day of lean beef based on a 2000-kcal diet; PSU, Penn State University.

Sixty-six participants were enrolled in the study. A total of 9 individuals withdrew from the study. Of those, 7 withdrew before completing the first diet period. The remaining 2 withdrew after diet period 1. Data from individuals who did not complete at least 1 full diet period were not included in analyses (*n* = 7). The main reason for withdrawing from the study was the inability to comply with the controlled feeding protocol due to social obligations. Participant recruitment for both sites is presented in **Figure 2**. The overall population of 59 participants (mean age ± SE: 49 ± 1.6 y; mean BMI ± SE: 27 ± 0.5) was healthy with multiple CVD risk markers within recommended ranges (mean ± SE LDL cholesterol: 109 ± 3.5 mg/dL) at the start of the study. Baseline participant characteristics are presented in **Table 2**.

**TABLE 2:** Characteristics of study participants at baseline (*n* = 59)^1^

	Mean ± SEM
Age, y	49 ± 1.6
Males:females, *n*	28:31
BMI, kg/m^2^	27 ± 0.5
TC, mg/dL	193 ± 4.8
LDL cholesterol, mg/dL	109 ± 3.5
HDL cholesterol, mg/dL	55 ± 1.9
TG, mg/dL	105 ± 7.9
Glucose, mg/dL	99 ± 1.1
SBP, mmHg	117 ± 1.7
DBP, mmHg	77 ± 1.2

^1^Baseline values were measured before consuming any study food. DBP, diastolic blood pressure; SBP, systolic blood pressure; TC, total cholesterol; TG, triglycerides.

### Lipids, lipoproteins, and apolipoproteins

#### Endpoint to endpoint mean comparisons

The results of endpoint to endpoint analyses showed that total cholesterol, non-HDL cholesterol, and LDL cholesterol, were significantly lower following the MED0.5, MED2.5, and MED5.5 compared with the AAD (*P* < 0.0001); no differences were observed between the MED diets (**Table 3**). There were no differences in HDL cholesterol following consumption of all test diets. A dose–response effect was not detected for increasing lean beef dose on total cholesterol, LDL cholesterol, HDL cholesterol, non-HDL cholesterol, or triglycerides.

**TABLE 3:** Lipid, lipoprotein, and apolipoprotein concentrations after 4 wk of consuming each test diet^1^

Outcome	Baseline	AAD	MED0.5	MED2.5	MED5.5	Diet main effect *P* value
TC, mg/dL	192.6 ± 4.8	185.0 ± 4.4^a^	172.8 ± 4.1^b^	174.4 ± 4.4^b^	178.3 ± 3.9^b^	<0.0001^†^
Non-HDL cholesterol, mg/dL	137.5 ± 4.6	133.5 ± 4.4^a^	123.0 ± 4.1^b^	124.1 ± 4.3^b^	126.9 ± 3.9^b^	<0.0001^†^
LDL cholesterol, mg/dL	109.4 ± 3.5	108.5 ± 3.8^a^	98.7 ± 3.5^b^	99.8 ± 3.8^b^	102.0 ± 3.2^b^	<0.0001^†^
HDL cholesterol, mg/dL	55.0 ± 1.9	51.6 ± 1.5	49.8 ± 1.5	50.3 ± 1.5	51.4 ± 1.5	0.0341
TG, mg/dL	105.4 ± 7.9	92.9 ± 5.6	94.2 ± 5.5	93.9 ± 6.4	88.5 ± 5.3	0.0437
apoA1, mg/dL	148.5 ± 3.1	139.7 ± 2.7	136.7 ± 2.5	136.8 ± 2.5	140.0 ± 2.6	0.0034
apoB, mg/dL	94.4 ± 2.8	91.4 ± 2.7^a^	85.5 ± 2.5^b^	85.8 ± 2.7^b^	87.8 ± 2.4^b^	<0.0001^†^

^1^All values are means ± SEMs (*n* = 59). The MIXED procedure (version 9.4; SAS Institute Inc.) was used to test the effects of diet. Values in the same row with different superscript letters are significantly different (Tukey-adjusted *P* < 0.05). ^†^Value is statistically significant, *P* < 0.003 (Bonferroni adjusted α for multiple comparisons). AAD, average American diet; MED, Mediterranean-style eating pattern used in the study; MED0.5, MED diet with 14 g (0.5 oz) per day of lean beef; MED2.5, MED diet with 71 g (2.5 oz) per day of lean beef; MED5.5, MED diet with 156 g (5.5 oz) per day of lean beef based on a 2000-kcal diet; TC, total cholesterol; TG, triglycerides.

There were greater reductions in total LDL particle number (*P* < 0.003) and large LDL particles (*P* < 0.05) following the MED0.5 and MED2.5 compared with the AAD (**Table 4**). The reduction in particle number following the MED5.5 was not different from the other MED diets or AAD; however, the reduction in large LDL particles compared with the MED0.5 was significantly less. There were no diet effects for IDLs or small LDLs. A dose–response effect was not observed (data not shown).

**TABLE 4:** Lipid subparticle concentrations after 4 wk of consuming each test diet^1^

Endpoint	Baseline	AAD	MED0.5	MED2.5	MED5.5	Diet main effect *P* value
LDL particle number (total), nmol/L	1096.4 ± 43.6	1050.5 ± 39.6^a^	968.3 ± 37.6^b^	970.0 ± 37.8^b^	1022.9 ± 33.5^ab^	0.0002^†^
Large LDL particles, nmol/L	427.9 ± 21.0	402.8 ± 22.4^a^	327.2 ± 22.7^b^	360.3 ± 23.8^bc^	398.0 ± 21.5^ac^	<0.0001^†^
IDL, nmol/L	228.8 ± 18.3	189.9 ± 14.6	177.4 ± 12.1	163.7 ± 10.5	172.7 ± 14.3	0.3579
Small LDL particles, nmol/L	439.7 ± 38.6	457.8 ± 33.7	463.7 ± 32.4	446.2 ± 31.8	452.3 ± 35.4	0.8236
LDL size, nm	21.1 ± 0.1	21.1 ± 0.1^a^	20.9 ± 0.1^b^	20.9 ± 0.1^ab^	21.0 ± 0.1^ab^	0.0016^†^
HDL particle number (total), μmol/L	34.6 ± 0.6	32.8 ± 0.6	32.6 ± 0.6	32.5 ± 0.5	33.4 ± 0.6	0.0359
Large HDL particles, μmol/L	8.2 ± 0.5	7.8 ± 0.5	7.9 ± 0.4	7.9 ± 0.4	7.7 ± 0.4	0.7394
Medium HDL particles, μmol/L	11.2 ± 0.6	10.9 ± 0.6	10.3 ± 0.6	11.1 ± 0.6	11.4 ± 0.6	0.1428
Small HDL particles, μmol/L	15.3 ± 0.8	14.0 ± 0.9	14.5 ± 0.7	13.8 ± 0.7	14.2 ± 0.8	0.6904
HDL size, nm	9.5 ± 0.1	9.5 ± 0.1	9.5 ± 0.1	9.5 ± 0.1	9.5 ± 0.1	0.3606
VLDL, chylomicron, TG particle concentration, mg/dL	76.2 ± 6.6	66.4 ± 4.6	67.8 ± 4.2	66.3 ± 4.7	60.6 ± 4.0	0.0261

^1^All values are means ± SEMs (*n* = 59). The MIXED procedure (version 9.4; SAS Institute, Inc.) was used to test the effects of diet. Values in the same row with different superscript letters are significantly different (Tukey-adjusted *P* < 0.05). ^†^Value is statistically significant, *P* < 0.003 (Bonferroni adjusted α for multiple outcomes). AAD, average American diet; MED, Mediterranean-style eating pattern used in the study; MED0.5, MED diet with 14 g (0.5 oz) per day of lean beef; MED2.5, MED diet with 71 g (2.5 oz) per day of lean beef; MED5.5, MED diet with 156 g (5.5 oz) per day of lean beef based on a 2000-kcal diet; TG, triglyceride.

There were no diet effects for the number of large HDL particles, medium HDL particles, or small HDL particles (Table 4). Following the MED5.5 total HDL particle number was greater than following the MED0.5 (33.4; 95% CI: 32.6, 34.2; compared with 32.6; 95% CI: 31.7, 33.3; *P* < 0.05, respectively). A dose–response analysis confirmed this diet effect. In addition, the analysis revealed a greater reduction in medium HDL particles following the MED0.5 compared with the MED5.5 (*P* < 0.05). However, the reductions following MED2.5 were not significantly different from MED0.5 or MED5.5. Dose–response analysis data are not shown.

Diet effects on apoB reflected lipoprotein changes; the MED0.5, MED2.5, and MED5.5 decreased apoB by −6.3 (95% CI: −3.4, −9.7; *P* < 0.001), −5.9 (95% CI: −2.6, −9.1; *P* < 0.001), and −3.9 (95% CI: −0.7, −7.2; *P* < 0.01) compared with the AAD, respectively. There was no difference in apoB after consumption of the 3 MED diets. Reductions in apoA1 were not different between diets (*P* > 0.003). A dose–response analysis showed that when compared with the MED diets with low (MED0.5) and moderate (MED2.5) amounts of lean beef, the MED5.5 attenuated the reduction in apoA1 observed in the other 2 MED diet groups (*P* < 0.05 for both). No effect of dose was found for apoB (data not shown).

#### Change from baseline

**FIGURE 3:**
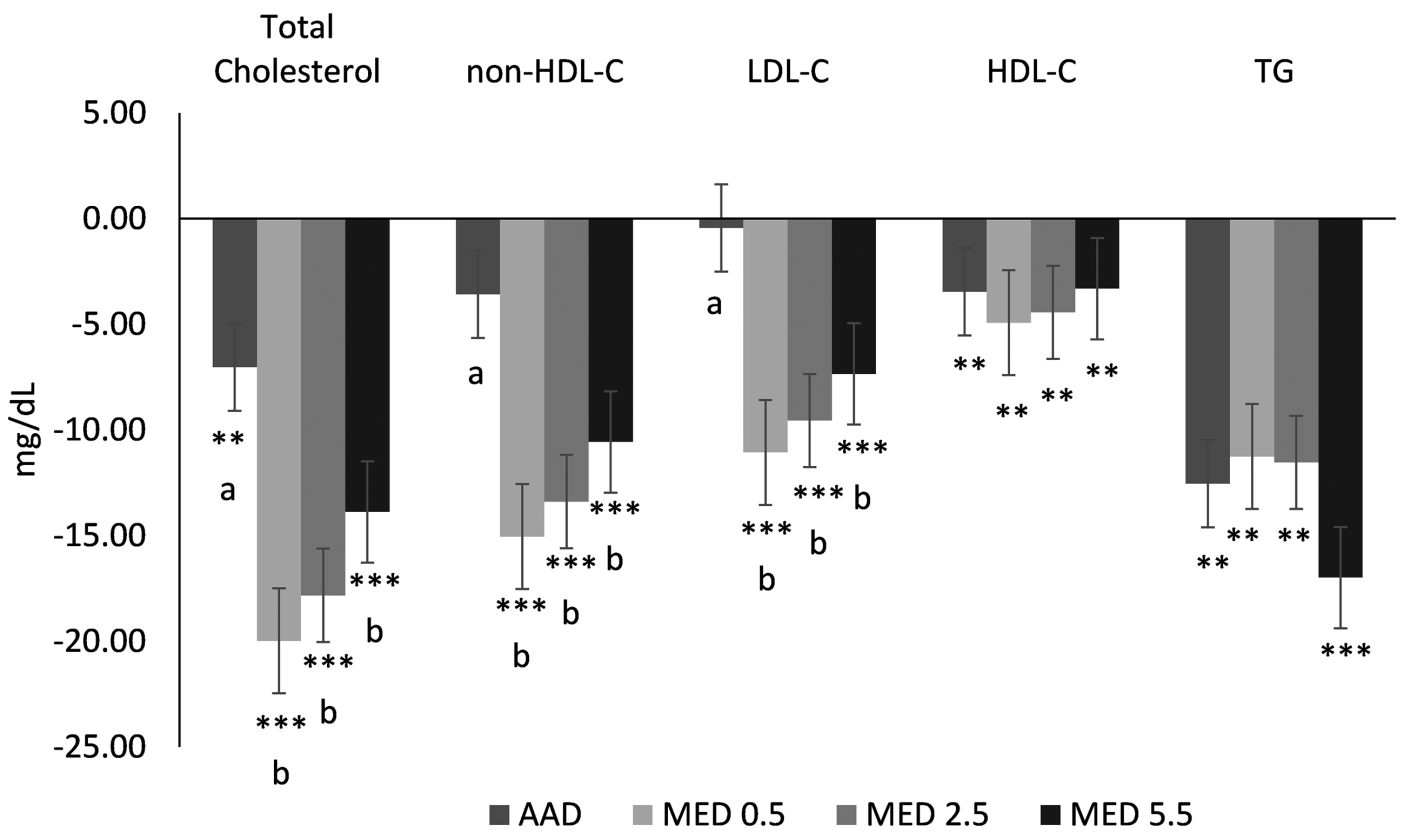
Change from baseline in lipids and lipoproteins after 4 wk of consuming each test diet. Mean change (±SEM) from baseline (*n* = 59). The MIXED procedure in SAS (version 9.4; SAS Institute Inc.) was used to test for within- and between-diet effects. ^*,**,***^Significantly different from baseline: *P < 0.05, **P < 0.01, ***P < 0.001. Where the main effect for diet was statistically significant at a value of *P* < 0.003 (Bonferroni adjusted α for multiple comparisons), post hoc testing was conducted and different letters are significantly different, *P* ≤ 0.01. AAD, average American diet; HDL-C, HDL cholesterol; LDL-C, LDL cholesterol; MED, Mediterranean-style eating pattern used in the study; MED0.5, MED diet with 14 g (0.5 oz) per day of lean beef; MED2.5, MED diet with 71 g (2.5 oz) per day of lean beef; MED5.5, MED diet with 156 g (5.5 oz) per day of lean beef based on a 2000-kcal diet; non-HDL-C, non-HDL cholesterol; TG, triglycerides.

**FIGURE 4:**
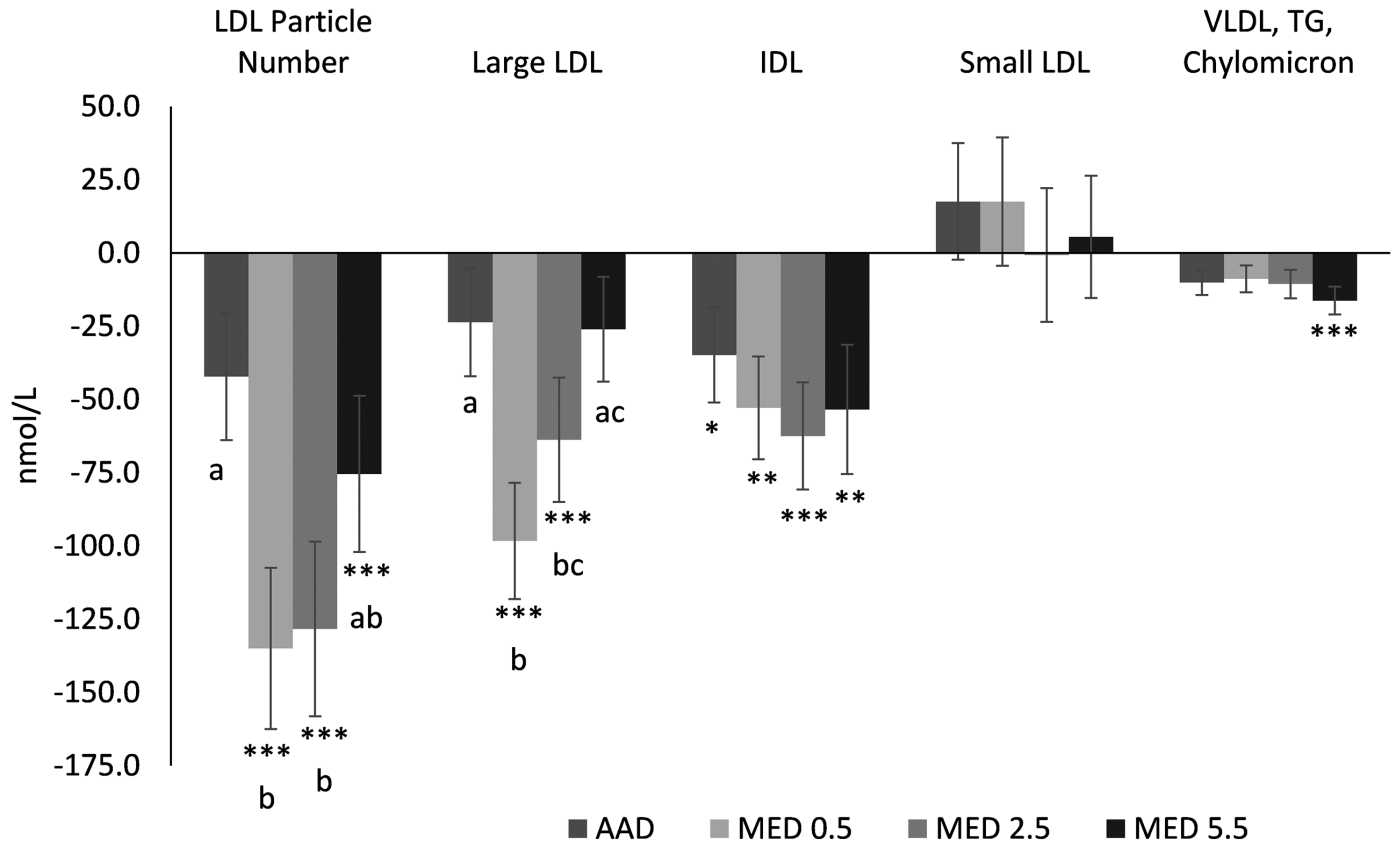
Change from baseline in non-HDL subparticles after 4 wk of consuming each test diet. Mean change (±SEM) from baseline (*n* = 59). The MIXED procedure in SAS (version 9.4; SAS Institute Inc.) was used to test for within- and between-diet effects. ^*,**,***^Significantly different from baseline: *P < 0.05, **P < 0.01, ***P < 0.001. Where the main effect for diet was statistically significant at a value of *P* < 0.003 (Bonferroni adjusted α for multiple comparisons), post hoc testing was conducted and different letters are significantly different, *P* ≤ 0.05. AAD, average American diet; MED, Mediterranean-style eating pattern used in the study; MED0.5, MED diet with 14 g (0.5 oz) per day of lean beef; MED2.5, MED diet with 71 g (2.5 oz) per day of lean beef; MED5.5, MED diet with 156 g (5.5 oz) per day of lean beef based on a 2000-kcal diet; TG, triglycerides.

**FIGURE 5:**
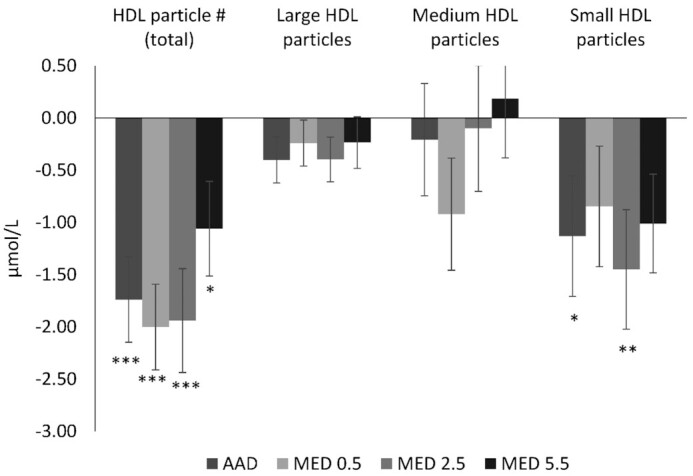
Change from baseline in HDL subclasses after 4 wk of consuming each test diet. Mean change (±SEM) from baseline (*n* = 59). The MIXED procedure in SAS (version 9.4; SAS Institute Inc.) was used to test for within- and between-diet effects. #, number. ^*,**,***^Significantly different from baseline: *P < 0.05, **P < 0.01, ***P < 0.001. AAD, average American diet; MED, Mediterranean-style eating pattern used in the study; MED0.5, MED diet with 14 g (0.5 oz) per day of lean beef; MED2.5, MED diet with 71 g (2.5 oz) per day of lean beef; MED5.5, MED diet with 156 g (5.5 oz) per day of lean beef based on a 2000-kcal diet.

**FIGURE 6:**
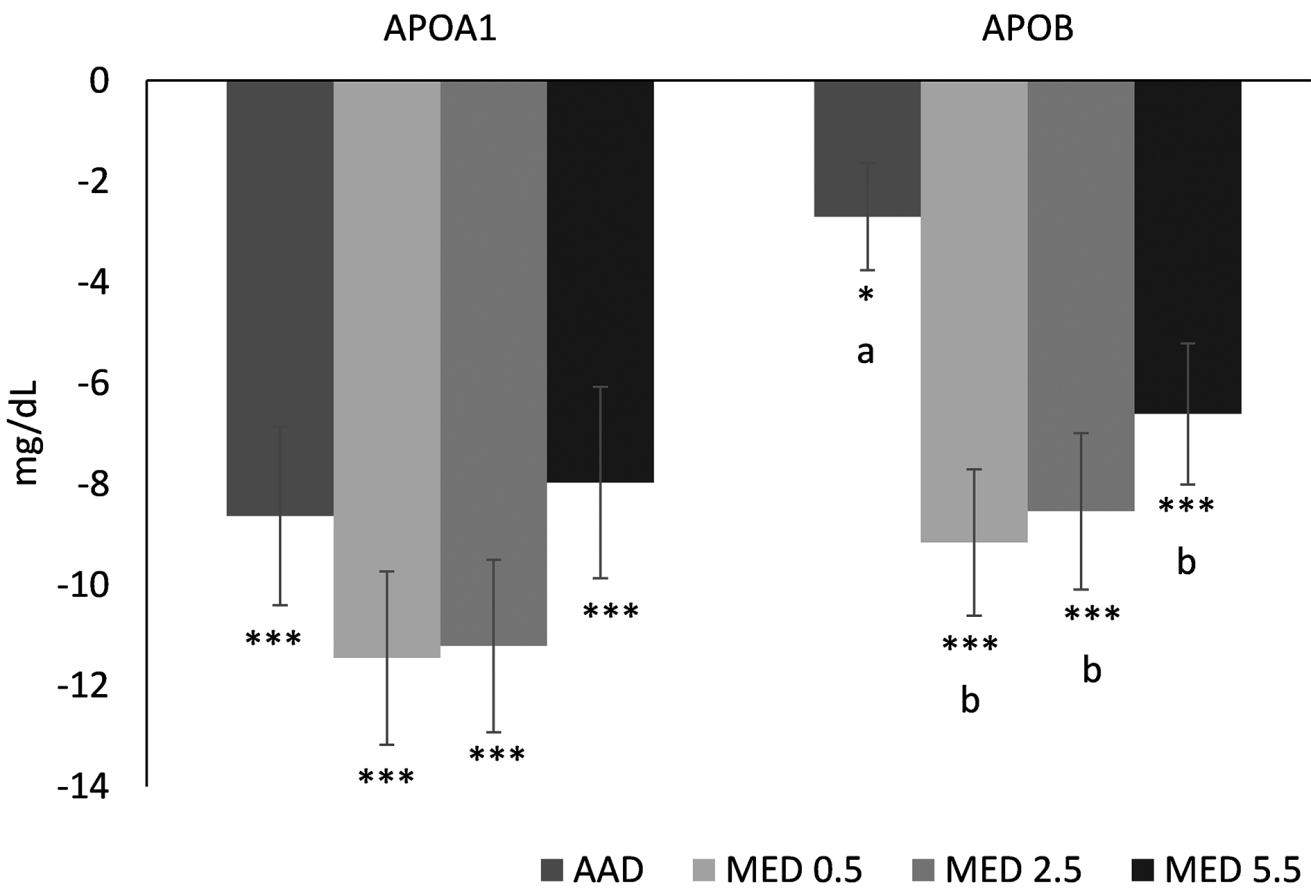
Change from baseline in apolipoproteins after 4 wk of consuming each test diet. Mean change (±SEM) from baseline (*n* = 59). The MIXED procedure in SAS (version 9.4; SAS Institute Inc.) was used to test for within- and between-diet effects. ^*,**,***^Significantly different from baseline: *P < 0.05, **P < 0.01, ***P < 0.001. Where the main effect for diet was statistically significant at a value of *P* < 0.003 (Bonferroni adjusted α for multiple comparisons), post hoc testing was conducted and different letters are significantly different, *P* ≤ 0.01. AAD, average American diet; MED, Mediterranean-style eating pattern used in the study; MED0.5, MED diet with 14 g (0.5 oz) per day of lean beef; MED2.5, MED diet with 71 g (2.5 oz) per day of lean beef; MED5.5, MED diet with 156 g (5.5 oz) per day of lean beef based on a 2000-kcal diet.

There was a significant reduction from baseline in LDL cholesterol for all 3 MED diets (*P* < 0.0001). Compared with the AAD, LDL cholesterol was reduced by −10.3 mg/dL (95% CI: −5.4, −15.7 mg/dL; *P* < 0.001), −9.1 mg/dL (95% CI: −3.9, −14.3 mg/dL; *P* < 0.001), and −6.9 mg/dL (95% CI: −1.7, −12.1 mg/dL; *P* < 0.005) with the MED0.5, MED2.5, and MED5.5 diets, respectively (**Figure 3**). Total cholesterol was lower following MED0.5, MED2.5, and MED5.5 diets by −12.8 mg/dL (95% CI: −6.8, −19.0 mg/dL; *P* < 0.001), −10.9 mg/dL (95% CI: −4.8, −17.0 mg/dL; *P* < 0.001), and −6.9 mg/dL (95% CI: −0.8, −13.0 mg/dL; *P* < 0.05) respectively, compared with the AAD. Non-HDL cholesterol was decreased on the MED0.5, MED2.5, and MED5.5 diets by −11.2 mg/dL (95% CI: −5.8, −16.9 mg/dL; *P* < 0.001), −9.8 mg/dL (95% CI: −4.2, −15.4 mg/dL; *P* < 0.001), and −7.0 mg/dL (95% CI: −1.4, −12.6 mg/dL; *P* < 0.01), respectively, compared with the AAD (Figure 3). All diets decreased triglycerides from baseline (*P* < 0.01). Compared with baseline, all MED diets significantly reduced LDL particle number (*P* < 0.0001) with greater reductions in LDL particle number for the MED0.5 (−91.2 nmol/L; 95% CI: −31.4, −151.0 nmol/L) and MED2.5 (−85.3 nmol/L; 95% CI: −25.4, −145.2 nmol/L) compared with the AAD (*P* < 0.003) (**Figure 4**). All diets were associated with reductions in HDL particle number when compared with baseline (**Figure 5**). There was a greater reduction from baseline in apoB for all 3 MED diets compared with the AAD (*P* < 0.01) (**Figure 6**).

### PCSK9

**FIGURE 7:**
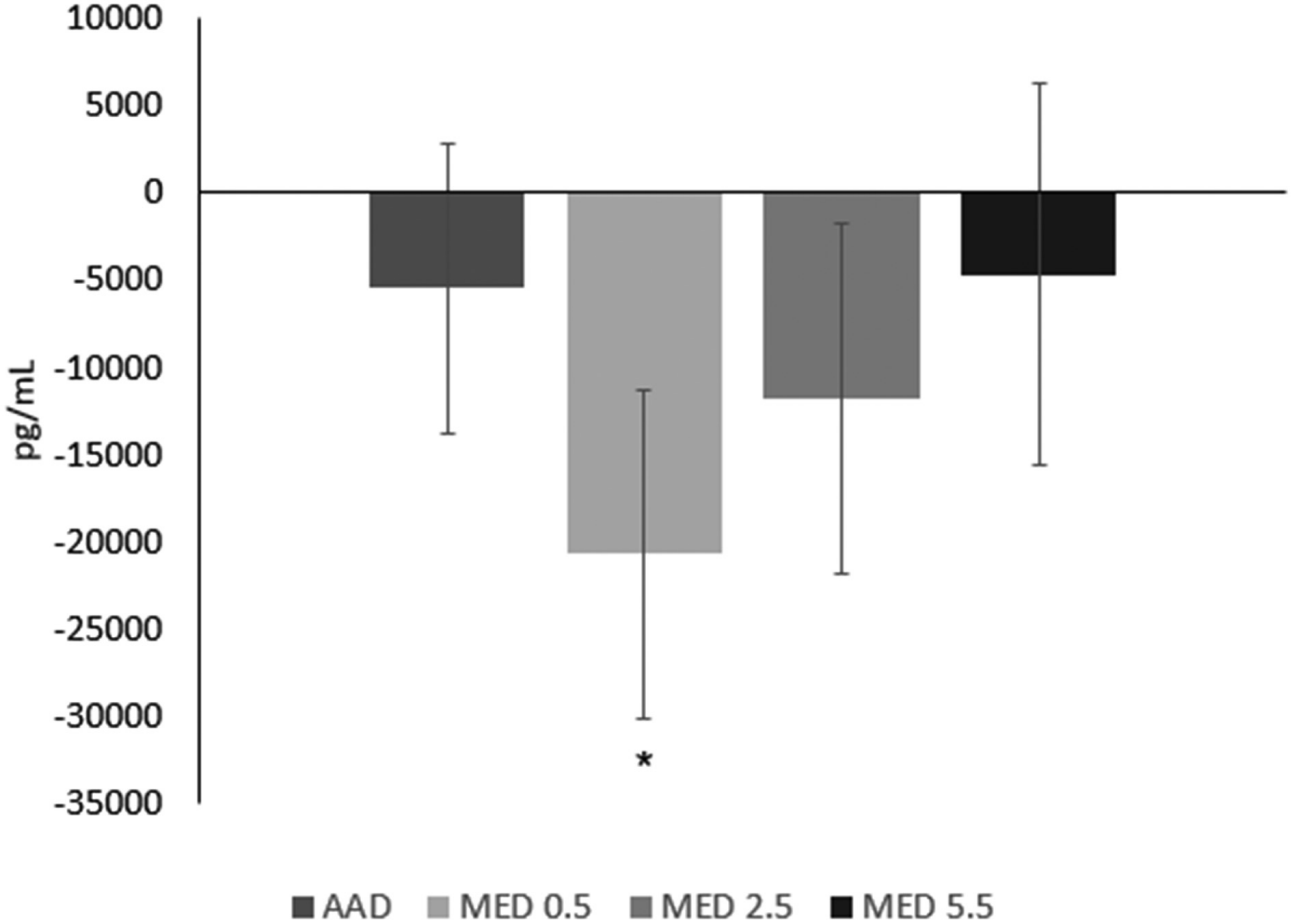
Change from baseline in plasma proprotein convertase subtilisin/kexin type 9 after 4 wk of consuming each test diet. Mean change (±SEM) from baseline (*n* = 59). The MIXED procedure in SAS (version 9.4; SAS Institute Inc.) was used to test for within- and between-diet effects. *Significantly different from baseline, *P < 0.05. AAD, average American diet; MED, Mediterranean-style eating pattern used in the study; MED0.5, MED diet with 14 g (0.5 oz) per day of lean beef; MED2.5, MED diet with 71 g (2.5 oz) per day of lean beef; MED5.5, MED diet with 156 g (5.5 oz) per day of lean beef based on a 2000-kcal diet.

There were no differences in PCSK9 after consumption of all test diets. All diets (MED 0.5, 2.5, and 5.5) as well as the AAD elicited a slight downward response in PCSK9 concentrations when compared with baseline with only the reduction in the MED0.5 reaching a *P* value <0.05 (**Figure 7**).

### Dietary analysis

Based on an evaluation of the test diets against the 14-point Mediterranean Diet Assessment Scale (16), there was a substantial point differentiation between the MED0.5 (12 points) and MED5.5 (7 points) as a result of the increase in lean beef (**Table 5**). Specifically, as the quantity of lean beef increased there was a reduction in the servings of nuts, legumes, and fish. Based on findings from the Prevención con Dieta Mediterránea (PREDIMED) study a score of ≥10 points corresponds to high dietary adherence. In the present study, both the MED0.5 (12 points) and MED2.5 (10 points) met the definition for high adherence to a Mediterranean dietary pattern, while also eliciting the greatest cardiovascular benefit (compared with the MED5.5) when compared with the AAD.

**TABLE 5:** Mediterranean diet adherence score of each test diet^1^

Component	AAD	MED0.5	MED2.5	MED5.5	PREDIMED
Olive oil main fat	0	1	1	1	1
Olive oil (≥4 tbsp)	0	1	1	1	1
Vegetables ≥2 svg/d (svg = 200 g)	0	1	1	1	1
Fruits ≥3 servings/d	1	1	1	1	1
Red or processed meats <100–150 g/d	1	1	1	0	1
Butter, cream, margarine <12 g/d	1	1	1	1	1
Soda drinks <1/d	1	1	1	1	1
Wine glasses ≥7/wk	0	0	0	0	1
Legumes ≥3 svg/wk (svg = 150 g)	0	1	1	0	1
Fish/seafood ≥3 svg/wk (svg = 100–150 g)	1	1	0	0	1
Commercial bakery ≤2/wk	1	1	1	1	1
Nuts ≥3 svg/wk (svg = 30 g)	0	1	1	0	1/0^1^
Poultry more than red meats	0	1	0	0	1
Use of sofrito sauce ≥2/wk	0	0	0	0	1
TOTAL SCORE:	6	12	10	7	11/12

1 Based on a 14-item dietary questionnaire used in the PREDIMED study to assess adherence to the Mediterranean diet (19). These numbers represent each of the 2 MedDiets provided in the PREDIMED Study. The 1 represents the score for the MedDiet with nuts and the 0 represents the score for the MedDiet with olive oil. svg, serving; tbsp, tablespoon.

## Discussion

Our results demonstrate that the consumption of a healthy Mediterranean-style dietary pattern with different amounts of lean beef (14, 71, or 156 g/d), improves lipids and lipoproteins when compared with a typical American dietary pattern containing 71 g/d of lean beef. These findings are consistent with previous research showing that consuming lean, unprocessed red meat (≤156 g beef/d/2000 kcal) as part of a DASH-style diet does not attenuate the favorable effects on lipids and lipoproteins (4). Similar findings also were observed with the inclusion of lean beef and pork (500 g/wk) as part of a Mediterranean-style diet compared with a Mediterranean diet containing 200 g/wk of beef or pork (8).

The Mediterranean Diet, 1 of 3 Healthy Eating Patterns recommended in both 2020–2025 (17) and the 2015–2020 Dietary Guidelines, includes ∼12.5 oz protein equivalents per week (50 g/d) of red meat (18), with an emphasis on lean cuts. To put this in perspective, the traditional Mediterranean diet of the 1960s included >200 g/d of meat in certain regions of the Mediterranean (13); the participants consuming a Mediterranean diet as part of the PREDIMED study reported an average of 120 g (4 oz)/d of meat and meat products (19), whereas current US intake is ∼71 g (2.5 oz)/d (10). Given the cardiovascular benefits of a traditional Mediterranean-style diet, this suggests the adoption of a dietary pattern abundant in nutrient-dense plant foods high in antioxidants and polyphenolic compounds, as well as other bioactives, will allow for consumption of low to moderate (≤71 g/d) quantities of lean beef.

In our study, all 3 MED diets elicited significant reductions from baseline in both LDL cholesterol concentration and LDL particle number. Moreover, a dose–response analysis revealed no attenuation of the LDL-lowering response with increasing quantities of lean beef when incorporated into a MED diet. When compared with the AAD control diet only the MED0.5 and MED2.5 elicited greater reductions in total LDL particle number and large LDL particle number. With lower PCSK9 concentrations being associated with greater removal of LDL particles from circulation (20) it is possible that the reduction in PCSK9 could have contributed to the decrease in LDL particle number. Although it remains unclear why PCSK9 was reduced to a greater magnitude relative to baseline with the MED0.5, despite a similar reduction in LDL particle number by the MED2.5, a nonsignificant downward trend in PCSK9 in the MED2.5 was observed. As an exploratory endpoint it is likely that this study was underpowered to detect a significant effect in PCSK9, thus this remains an area that warrants further investigation. Consistent with reductions observed for LDL cholesterol, all 3 MED diets also elicited greater reductions in apoB compared with the AAD. Richard et al. (21) reported similar decreases in plasma apoB in participants following a Mediterranean diet for 5 wk under controlled feeding conditions.

One plausible explanation for the modest differences in magnitude of LDL particle lowering among the 3 MED diets might be a result of the food replacement strategies used when increasing amounts of lean beef were added to the diet. Damasceno et al. (22) demonstrated that the greatest reductions in both LDL cholesterol concentration and LDL particle number were observed with the inclusion of nuts as part of a Mediterranean diet. In the present study, there was a considerable reduction in nuts and legumes in the MED5.5 compared with the MED0.5 and 2.5 to compensate for the increase in lean beef. Thus, the replacement strategy used for the MED5.5 could have contributed to the LDL particle number being no different from the AAD. Our results reflect changes in the dietary pattern rather than inclusion of a single food (lean beef). This illustrates the importance of establishing a healthy Mediterranean dietary pattern that embodies balance, variety, and the inclusion of all nutrient-rich components, which can include lean beef in moderation.

All diets were associated with reductions in HDL cholesterol concentration and HDL particle number when compared with baseline. A reduction in HDL particle size has been shown to be positively associated with CVD (23), and a greater number of small HDL particles are associated with increased CVD risk in healthy adults (24).

Large, spherical HDLs, in contrast, are inversely correlated with CVD risk (23) and are considered to be the preferred acceptors of the cholesterol that effluxes from macrophages and are modulated by the (ATP binding cassette transporter G1) ABCG1-mediated pathway (25). In the present study, the reduction in HDL concentration and particle number appears to be driven by the loss of small HDL particles, which elicited the greatest reduction from baseline following the AAD and MED2.5. The observed reductions in apoA1 are consistent with the reductions in HDL particle number. In a study of cynomolgus monkeys, the isocaloric substitution of dietary SFAs with either MUFAs or PUFAs significantly reduced plasma HDL and apoA1 concentrations due to enhanced apoA1 catabolism (26). That is, apoA1 is cleared at a faster rate during consumption of a high-MUFA diet relative to a high-SFA diet. Similarly, Richard et al. (27) found reductions in apoA1 concentration and production rate following a reduction in SFAs as part of a Mediterranean diet. They hypothesized that the significant concomitant decrease in LDL cholesterol and apoB decreased the need for reverse cholesterol transport. Research into the role of HDL functionality in cardiovascular disease is ongoing (28).

A major strength of our study is the randomized controlled crossover design and low dropout rate (<15%). High levels of dietary compliance were attained as verified by the completion of daily and weekly monitoring forms. To our knowledge, this is the first study to examine the effects of a Mediterranean diet pattern containing 3 levels of lean red meat on lipids, lipoproteins, and apolipoproteins in a US population. Additionally, our population was generally healthy, with near optimal LDL cholesterol concentrations, which makes our findings more relevant and generalizable. Future research should investigate these effects of diet in a less healthy population. Limitations of the study include the lack of biological measures of compliance and sole reliance on partial observation and self-reported measures of adherence. Although the participants received all of their foods using a controlled feeding design, which provides “very tight” diet control, they were not blinded to the dietary treatments. In designing the treatment diets we chose to include a MED diet with the traditional amount of red meat (14 g/d); however, it is unclear how the results might have differed if a diet containing no red meat was examined. Moreover, the dietary substitutions made to incorporate red meat impacted the whole dietary pattern, particularly the MED5.5 diet, where the Mediterranean Diet Assessment Scale score reflected low adherence (<10 points) to a Mediterranean diet (because lean beef isocalorically replaced nuts, legumes, and fish) (19). Thus, we cannot determine whether the inclusion of lean red meat or the reduction in adherence to a Mediterranean diet contributed to the observed differences in lipids and lipoproteins between the MED0.5 and MED5.5. Finally, although unintentional, our study population was a sample of predominantly Caucasian individuals, thus limiting the generalizability to other races and ethnicities. However, there is evidence from the DELTA study to show that diet effects are remarkably similar across different population groups, which suggests that diet can have a significant impact on risk for cardiovascular disease in the total population (29).

In conclusion, consumption of healthy Mediterranean-style dietary patterns containing different amounts of lean beef (14, 71, or 156 g/d) improved lipids and lipoproteins compared with a typical American dietary pattern. Notably, the benefits of a healthy, low saturated fat, Mediterranean-style diet were not attenuated by the inclusion of small to moderate amounts of lean beef. This exemplifies the contribution of the portfolio of healthy foods in a Mediterranean-style diet to the lipid and lipoprotein benefits compared with the AAD. With MED diets containing 14, 71, 156 g/d of lean beef we observed significant lowering of total cholesterol, LDL cholesterol, non-HDL cholesterol, and apoB compared with a typical American-style diet. LDL particle number was lowered to a greater extent with MED0.5 (low) and 2.5 (moderate), suggesting greater CVD risk reduction with low to moderate amounts of lean beef incorporated in the diet when compared with similar amounts of lean beef included in the AAD. These findings are consistent with the transition to dietary pattern-based recommendations and demonstrate that lean beef in amounts ≤71 g (2.5 oz)/d can be part of a healthy Mediterranean-style dietary pattern without attenuating the cardiovascular benefits.

## Supplementary Material

nqaa375_Supplemental_File

## Notes

This study was funded by the Beef Checkoff. This study also was supported by the USDA, ARS, and the Penn State Clinical and Translational Research Institute, Pennsylvania State University Clinical and Translational Science Award, and NIH/National Center for Advancing Translational Sciences grant no. UL1TR000127.

The financial supporters had no role in the design and conduct of the study, collection, analysis, and interpretation of data, or preparation, review, or approval of the manuscript.

Data described in the manuscript, code book, and analytic code will be made available upon request following publication.

Abbreviations used: AAD, average American diet; ALA, α-linolenic acid (18:3n–3); CVD, cardiovascular disease; DASH, Dietary Approaches to Stop Hypertension Trial; MED, Mediterranean-style eating pattern used in the study; MED0.5, Mediterranean-style eating pattern with 14 g (0.5 oz) lean beef per day; MED2.5, Mediterranean-style eating pattern with 71 g (2.5 oz) lean beef per day; MED5.5, Mediterranean-style eating pattern with 156 g (5.5 oz) lean beef per day; PCSK9, plasma proprotein convertase subtilisin/kexin type 9; PREDIMED, Prevención con Dieta Mediterránea; TC, total cholesterol.
