# Supplementary material for: Effect of varying quantities of lean beef as part of a Mediterranean-style dietary pattern on lipids and lipoproteins: a randomized crossover controlled feeding trial
Source: Am J Clin Nutr. 2021 Apr 7;113(5):1126–36. doi: 10.1093/ajcn/nqaa375 (PMC8106750; doi:10.1093/ajcn/nqaa375)
Supplement: nqaa375_Supplemental_File [file nqaa375_supplemental_file.docx]

**Supplementary Table 1. Food Based Dietary Pattern Comparison Based on ~2000 kcal Diet**

|  | Traditional Mediterranean Diet^1^ | | **MED 0.5**  goal/day | **MED 5.5**  goal/day | **AAD^2^** |
| --- | --- | --- | --- | --- | --- |
|  | Goal as listed in pyramid | Goal/day |  |  |  |
| Grain servings | 1-2/meal | 3-6 | **4** | **4** | 6.4 oz/day |
| Whole grain servings | Preferably whole grain | Preferably whole grain |  |  | 0.6 oz/day |
| Fruit servings | 1-2/meal | 3-6 | **4** | **3** | 1.0 c/day |
| Vegetable servings | ≥ 2/meal | ≥ 6 | **6** | **6** | 1.6 c/day |
| Olive Oil (1 tsp = 1 svg) | 1-2/meal | 3-6 | **6** | **6** | 18 g/day**^3^** |
| Olives/Nuts/Seed servings | 1-2/day | 1-2 | **1** | **1** | 0.5 oz/day |
| Dairy servings | 2/day | 2 | **2** | **2** | 1.5 c/day |
| Sweets | ≤ 2/week | ≤ 0.3 |  |  |  |
| Potato servings | ≤ 3/week | ≤ 0.4 | **≤ 0.4** | **≤ 0.4** |  |
| Protein servings |  |  | **7 oz** | **7 oz** |  |
| Eggs | 2-4/week | 0.3-0.6 | **1** | **1** | 0.4 oz/day |
| Red meat | < 2/week | < 0.3 | **0.5 oz** | **5.5 oz** | 2.5 oz/day^4^ |
| Processed meat | ≤ 1/week | ≤ 0.1 |  |  |  |
| Legume | ≥ 2/week | ≥ 0.3 | **0.3** | **0.3** | 0.1 c/day |
| White meat | 2/week | 0.3 | **1** | **0** | 1.2 oz/day |
| Fish/Seafood | ≥ 2/week | ≥ 0.3 | **1** | **0.4** | 0.5 oz/day |

^1^From the Mediterranean Diet Foundation (Fundación Dieta Mediterránea, <https://dietamediterranea.com/nutricion-saludable-ejercicio-fisico/#pyramid>), serving sizes are based on frugality and local habits

^2^Source of AAD (Average American Diet): Usual U.S. Intake Adults, Dietary Guidelines for Americans 2010 (Table 5-1).

^3^Amount listed is for total oil (breakdown of types of oil not determined).

^4^Amount listed is for meat.

**Supplementary Table 2. Sample menu**

| MED 0.5 | MED 2.5 | MED 5.5 | AAD |
| --- | --- | --- | --- |
| **BREAKFAST**  Whole grain bread w/strawberry preserves  Egg beaters w/ spinach  Applesauce  Skim milk  Olive oil | **BREAKFAST**  Whole grain bread w/strawberry preserves  Egg beaters w/ spinach  Applesauce  Skim milk  Olive oil | **BREAKFAST**  Whole grain bread w/strawberry preserves  Egg beaters w/ spinach  Applesauce  Skim milk  Olive oil | **BREAKFAST**  English muffin  Egg beaters  Turkey sausage  Applesauce  Skim milk  Butter |
| **LUNCH**  Minestrone soup  **Falafel**  Salad w/feta cheese  Tzatziki sauce  Pita bread  Sautéed zucchini and squash  Olive oil | **LUNCH**  Minestrone soup  **Falafel**  Salad w/feta cheese  Tzatziki sauce  Pita bread  Sautéed zucchini and squash  Olive oil | **LUNCH**  Minestrone soup  **Braised beef**  Salad  Tzatziki sauce  Pita bread  Sautéed zucchini and squash  Olive oil | **LUNCH**  Chicken salad sandwich on whole grain white bread w/ lettuce and tomato  Baby carrots  Pretzels  Brownie |
| **DINNER**  Ratatouille  Jasmine rice  **Baked cod**  Pita bread  Olive oil | **DINNER**  Ratatouille  Jasmine rice  **Lean beef**  Pita bread  Olive oil | **DINNER**  Ratatouille  Jasmine rice  **Lean beef**  Pita bread  Olive oil | **DINNER**  Ratatouille  Jasmine rice  Baked cod  Pita bread  Sunflower oil |
| **SNACK**  Greek yogurt  Peaches  **Almonds** | **SNACK**  Greek yogurt  Peaches  **Almonds** | **SNACK**  Greek yogurt  Peaches | **SNACK**  Red pepper dip  Pita bread  Cucumber |

Bold-faced foods indicate the differences across the three Mediterranean Diets.
